# New applications of the existence of solutions for equilibrium equations with Neumann type boundary condition

**DOI:** 10.1186/s13660-017-1357-4

**Published:** 2017-04-22

**Authors:** Zhaoqi Ji, Tao Liu, Hong Tian, Tanriver Ülker

**Affiliations:** 1 College of Civil Engineering and Architecture, Shandong University of Science and Technology, Qingdao, 266590 China; 2 College of Environmental Science and Engineering, Ocean University of China, Qingdao, 266100 China; 3 College of Earth Science and Engineering, Shandong University of Science and Technology, Qingdao, 266590 China; 4 Departamento de Matemática, Universidad Austral, Paraguay 1950, Rosario, S2000FZF Argentina

**Keywords:** Neumann type boundary condition, set-valued measures, integral representation, topology

## Abstract

Using the existence of solutions for equilibrium equations with a Neumann type boundary condition as developed by Shi and Liao (J. Inequal. Appl. 2015:363, 2015), we obtain the Riesz integral representation for continuous linear maps associated with additive set-valued maps with values in the set of all closed bounded convex non-empty subsets of any Banach space, which are generalizations of integral representations for harmonic functions proved by Leng, Xu and Zhao (Comput. Math. Appl. 66:1-18, 2013). We also deduce the Riesz integral representation for set-valued maps, for the vector-valued maps of Diestel-Uhl and for the scalar-valued maps of Dunford-Schwartz.

## Introduction

The Riesz-Markov-Kakutani representation theorem states that, for every positive functional *L* on the space $C_{c}(T)$ of continuous compact supported functional on a locally compact Hausdorff space *T*, there exists a unique Borel regular measure *μ* on *T* such that $L(f) =\int f \,d\mu$ for all $f \in C_{c}(T)$. Riesz’s original form [3] was proved in 1909 for the unit interval $(T = [0; 1 ] )$. Successive extensions of this result were given, first by Markov in 1938 to some non-compact space (see [4]), by Radon for compact subset of $\mathbb{R}^{n}$ (see [5]), by Banach in note II of Saks’ book (see [6]) and by Kakutani in 1941 to a compact Hausdorff space [7]. Other extensions for locally compact spaces are due to Halmos [8], Hewith [9], Edward [10] and Bourbaki [11]. Singer [12, 13], Dinculeanu [14, 15] and Diestel-Uhl [16] gave an integral representation for functional on the space $C(T,E)$ of vector-valued continuous functions. Recently Leng, Xu and Zhao (see [2]) gave the integral representation for continuous functionals defined on the space $C(T)$ of all continuous real-valued functions on *T*; as an application, Shi and Liao (see [1]) also gave short solutions for the full and truncated *K*-moment problem. The set-valued measures, which are natural extensions of the classical vector measures, have been the subject of many theses. In the school of Pallu De La Barriere we have the ones of Thiam [17], Cost [18], Siggini [19], in the school of Castaing the one of Godet-Thobie [20], and in the school of Thiam the ones of Dia [21] and Thiam [22]. Investigations are undertaken for the generalization of results for set-valued measures in particular the Radon-Nikodym theorem for weak set-valued measures [2, 23] and the integral representation for additive strictly continuous set-values maps with regular set-valued measures. The work of Rupp in the two cases, *T* arbitrary non-empty set and *T* compact, allowed one to generalize the Riesz integral representation of additive and *σ*-additive scalar measures to the case of additive and *σ*-additive set-valued measures (see [24, 25]). He has proved among others that if *T* is a non-empty set and $\mathfrak{A}$ the algebra of subsets of *T*, for all continuous linear maps *l* defined on the space $\mathcal{B}(T;\mathbb{R})$ of all uniform limits of finite linear combinations of characteristic functions of sets in $\mathfrak{A}$ associated with an additive set-valued map with values in the space $\operatorname{ck}(\mathbb{R}^{n})$ of convex compact non-empty subsets of $\mathbb{R}_{n}$, there exists a unique bounded additive set-valued measure *M* from $\mathfrak{A}$ to the space $\operatorname{ck}(\mathbb{R}^{n})$ such that $\delta^{*}(\cdot |l(f))=\delta^{*}(\cdot |\int fM)$ and conversely. In this paper we extend this result to the case of any Banach space E. We deduce the Riesz integral representation for additive set-valued maps with values in the space of all closed bounded convex non-empty subsets of *E*; for vector-valued maps (see [16], Theorem 13, p.6) and for scalar-valued maps (see [26]).

## Notations and definitions

Let *E* be a Banach space and $E^{\prime}$ its dual space. We denote by $\Vert \cdot \Vert $ the norm on *E* and $E^{\prime}$. If *X* and *Y* are subsets of *E* we shall denote by $X + Y$ the family of all elements of the form $x + y$ with $x\in X$ and $y \in Y $, and by $X\,\dot{+}\,Y$ or $\operatorname {adh}(X + Y )$ the closure of $X + Y $. The closed convex hull of *X* is denoted by $\bar{\mathrm{co}}(X)$. The support function of *X* is the function $\delta^{*}(\cdot |X)$ from $E^{\prime}$ to $] - \infty;+\infty]$ defined by

$$\delta^{*}(y|X) = \sup \bigl\{ y(x); x \in X \bigr\} . $$

We denote by $\operatorname {cfb}(E)$ the set of all closed bounded convex non-empty subsets of *E*. We endowed $\operatorname {cfb}(E)$ with the Hausdorff distance denoted by *δ* and the structures $\dot{+}$ and the multiplication by positive real numbers. For all $K \in \operatorname {cfb}(E)$ and for all $K^{\prime}\in \operatorname {cfb}(E)$, we have

$$\delta\bigl(K;K^{\prime}\bigr) = \sup \bigl\{ \bigl\vert \delta^{*}(y|K)-\delta ^{*}\bigl(y|K^{\prime}\bigr) \bigr\vert ;y\in E\prime, \Vert y \Vert \le1 \bigr\} . $$

Recall that $(\operatorname {cfb}(E); \delta)$ is a complete metric space (see [27], Theorem 9, p.185). We denote by $C^{h}(E^{\prime})$ the space of all continuous real-valued map defined on $E^{\prime}$ and positively homogeneous. If $u \in C^{h}(E^{\prime})$, then we have

$$u(\lambda y) = \lambda u(y) $$

for all $y\in E^{\prime}$ and for all $\lambda\in\mathbb{R}$, where $\lambda\geq0$. We endowed $C^{h}(E^{\prime})$ with the norm

$$\Vert u \Vert = \sup \bigl\{ \bigl\vert u(y) \bigr\vert ; y \in E^{\prime}; \Vert y \Vert \le1 \bigr\} . $$

Put $C_{0} = \{ \delta^{*}(y|B); B \in \operatorname {cfb}(E) \} $ and put $\tilde{C}_{0} = C_{0} - C_{0}$; then $\tilde{C}_{0} $ is a subspace of the vector space $C^{h}(E^{\prime})$ generated by $C_{0}$. Let *T* be a non-empty set, let $\mathfrak{A}$ be an algebra consisting of subsets of *T* and let $B(T;\mathbb{R})$ be the space of all bounded real-valued functions defined on *T*, endowed with the topology of uniform convergence. We denote by $\mathcal{S}(T;\mathbb{R})$ the subspace of $\mathcal {B}(T;\mathbb{R})$ consisting of simple functions (*i.e.* of the form $\Sigma\alpha_{i}1_{A_{i}}$ where $\alpha_{i}\in\mathbb{R}$; $A_{i}\in\mathfrak{A}$;$\{ A_{1},A_{2},\cdot,A_{n} \}$ a partition of *A* and $1_{A_{i}}$ the characteristic function of $A_{i}$.) We denote by $B(T,\mathbb{R})$ the closure in $B(T;\mathbb{R})$ of $\mathcal{S}(T;\mathbb{R})$; $\mathcal{S}_{+}(T;\mathbb{R})$ (resp. $\mathcal{B}_{+}(T;\mathbb{R})$) the subspace of $\mathcal{S}(T;\mathbb{R})$ (resp. $\mathcal{B}(T;\mathbb{R})$) consisting of positive functions. We endowed $\mathcal{B}(T;\mathbb {R})$ with the induced topology. Notes that if $\mathfrak{A}$ is the Borel *σ*-algebra, then $\mathcal{B}(T;\mathbb{R})$ is the space of all bounded measurable real-valued functions. Let M be a set-valued map from $\mathfrak{A}$ to $\operatorname {cfb}(E)$. We say that *M* is additive if $M(\varnothing) = \{ 0 \}$ and

$$M(A \cup B) = M(A) \,\dot{+}\, M(B) $$

for all disjoint sets *A*, *B* in $\mathfrak{A}$. The set-valued measure *M* is said to be bounded if $\bigcup \{M(A), A \in\mathfrak{A} \}$ is a bounded subset of *E*. The semivariation of *M* is the map $\Vert M \Vert (\cdot)$ from $\mathfrak{A}$ to $[0;+\infty]$ defined by

$$\Vert M \Vert (A ) = \sup f \bigl\{ \bigl\vert \delta \bigl(y|M(\cdot)\bigr) \bigr\vert (A);y\in E^{\prime}, \Vert y \Vert \le1 \bigr\} , $$

where $\vert \delta(y|M(\cdot)) \vert (A)$ denotes the total variation of the scalar measure $\delta^{*}(y|M(\cdot))$ on *A* defined by

$$\biggl\vert \delta\bigl(y|M(\cdot)\bigr)|(A)=\sup \sum _{i}\delta^{*}\bigl(y|M(A_{i})\bigr) \biggr\vert ; $$

the supremum is taken over all finite partition $(Ai)$ of *A*; $A_{i} \in \mathfrak{A}$. If $\Vert M \Vert (T )<+\infty$, then *M* will be called a set-valued measure of finite semivariation. We denote by $\mathcal{M}(\mathfrak{A}; \operatorname {cfb}(E))$ the space of all bounded set-valued measures defined on $\mathfrak{A}$ with values in $\operatorname {cfb}(E)$. Let *m* be a vector measure from $\mathfrak{A}$ to *E*. We say that *m* is a bounded additive vector measure if its verifies similar conditions of bounded additive set-valued measures. We denote by $\Vert m \Vert $ the semivariation of *m* defined by $\Vert m \Vert (A) = \sup \{ \vert y\circ m \vert (A); y \in E^{\prime}; \Vert y \Vert \le1 \}$ where $\vert y\circ m \vert (A)$ denotes the total variation of the scalar measure $y\circ m$ on A defined by

$$\vert y\circ m \vert (A)=\sup \sum_{i} \bigl\vert y\bigl(m(A_{i})\bigr) \bigr\vert $$

for all $A \in\mathfrak{A}$; the supremum is taken over all finite partition $(Ai)$ of *A*; $A_{i} \in\mathfrak{A}$. Let $L: \mathcal{B}_{+}(T;\mathbb{R}) \rightarrow \operatorname {cfb}(E) $ be a set-valued map. We say that *L* is an additive (resp. positively homogeneous) if for all $f, g \in\mathcal{B}_{+}(T;\mathbb{R})$ (resp. for all $\lambda\ge0$), $L(f+g) = L(f)\,\dot{+}\,L(g)$ (resp. $L(\lambda f)=\lambda L(f)$). We denote by $\mathcal{L}(\mathcal {B}(T,\mathbb{R});C^{h}(E^{\prime}))$ the space of all linear continuous maps defined on $\mathcal{B}(T,\mathfrak{R})$ with values in $C^{h}(E^{\prime})$. If $l\in\mathcal{L}(\mathcal {B}(T,\mathbb{R});C^{h}(E^{\prime}))$; we put

$$\Vert l \Vert =\sup \bigl\{ \bigl\Vert l(f) \bigr\Vert ;f\in\mathcal {B}_{+}(T,\mathbb{R}), \Vert f \Vert \le1 \bigr\} , $$

where $\Vert f \Vert =\sup \{ \vert f(t) ; t\in T \vert \}$. For a numerical function *f* defined on *T*, we set $f^{+} = \sup (f, 0)$ and $f^{-} = \sup (-f, 0)$.

### Definition 2.1

Let $l\in\mathcal{L}(\mathcal{B}(T,\mathbb{R},C^{h}(E^{\prime})))$ and let $L:\mathcal{B}_{+}(T,\mathbb{R})\rightarrow \operatorname {cfb}(E)$ be an additive, positively homogeneous and continuous set-valued map. We say that *l* is associated with *L* if $l(f) = \delta^{*}(\cdot |L(f))$ for all $f\in\mathcal{B}_{+}(T;\mathbb{R})$. Then we have

$$l(f) = \delta^{*}\bigl(\cdot |L\bigl(f^{+}\bigr)\bigr)-\delta^{*}\bigl(\cdot |L\bigl(f^{-} \bigr)\bigr) \in\widetilde{C_{0}} $$

for all $f \in\mathcal{B}(T;\mathbb{R})$.

## Lemmas

In order to prove our main results, we need the following lemmas.

### Lemma 3.1

*Let* $M : \mathfrak{A}\rightarrow \operatorname {cfb}(E)$ *be an additive set*-*valued measure*. *Then* *M* *is bounded if and only if it is finite semivariation*.

### Proof

The set-valued measure *M* is bounded if there exists a nonnegative real number *c* such that

$$\sup_{A\in\mathfrak{A}}\sup_{ \Vert y \Vert \leq 1} \bigl\vert \delta^{*} \bigl(y|M(A)\bigr) \bigr\vert \leq c. $$

We have $\sup_{A\in\mathfrak{A}}\sup_{ \Vert y \Vert \leq1} \vert \delta^{*}(y|M(A)) \vert \leq\sup_{ \Vert y \Vert \leq1} \vert \delta^{*}(y|M(\cdot)) \vert (T)= \Vert M \Vert (T) $. On the other hand, by Lemma 5 (of [28], p.97) one has

$$\bigl\vert \delta^{*}\bigl(y|M(\cdot)\bigr) \bigr\vert (T)\leq2 \sup _{A\in\mathfrak {A}} \bigl\vert \delta^{*}\bigl(y|M(A)\bigr) \bigr\vert $$

for all $y \in E^{\prime}$. Then

$$\sup_{ \Vert y \Vert \leq1} \bigl\vert \delta^{*}\bigl(y|M(\cdot)\bigr) \bigr\vert (T)\leq2\sup_{A\in\mathfrak{A}}\sup_{ \Vert y \Vert \leq1} \bigl\vert \delta^{*}\bigl(y|M(A)\bigr) \bigr\vert . $$

Therefore

$$\sup_{A\in\mathfrak{A}}\sup_{ \Vert y \Vert \leq 1} \bigl\vert \delta^{*} \bigl(y|M(A)\bigr) \bigr\vert \leq\sup_{A\in\mathfrak {A}}\sup _{ \Vert y \Vert \leq1} \bigl\vert \delta ^{*}\bigl(y|M(A)\bigr) \bigr\vert . $$

 □

### Lemma 3.2

*Let* $C_{0}$ *be the set* $\{\delta^{*}(\cdot |B);B\in \operatorname {cfb}(E) \}$ *and let* $l : \mathcal{B}(T;\mathbb{R} )\rightarrow C^{h}(E^{\prime})$ *be a continuous linear map*. *Then* *l* *is associated with an additive*, *positively homogeneous and continuous set*-*valued map if and only if* $l(f) \in C_{0}$ *for all* $f \in\mathcal {B}_{+}(T,\mathbb{R})$.

### Proof

The necessary condition is obvious. Now assume that $l(f) \in C_{0}$ for all $f \in\mathcal{B}_{+}(T,\mathbb{R})$. Let consider the map $j : \operatorname {cfb}(E) \leftarrow C_{0}(B\to\delta^{*}(\cdot |B))$; then *j* is an isomorphism, more a homeomorphism (see [27], Theorem 8, p.185). Let $l^{\prime}$ be the restriction of *l* to $\mathcal{B}_{+}(T,\mathbb{R})$. If we put $L = j^{-1}\circ l^{\prime}$, then it is easy to see that *L* is additive, positively homogeneous and continuous. Therefore for all $f \in \mathcal{B}_{+}(T,\mathbb{R})$, we have

$$l(f) = \delta^{*}\bigl(\cdot |L(f)\bigr)\in C_{0}. $$

Let $M : \mathfrak{A}\rightarrow \operatorname {cfb}(E)$ be a bounded additive set-valued measure. For all $h \in\mathcal{S}_{+}(T,\mathbb{R})$ such that $h =\sum a_{i}1_{B_{i}}$ and for all $A \in\mathfrak{ A}$, the integral $\int_{A} hM $ of *h* with respect to *M* is defined by $\int_{A} hM =\operatorname {adh}(a_{1}M(A\cap B_{1})+a_{2}M(A\cap B_{2})+\cdots +a_{n}M(A\cap B_{n}))$. This integral is uniquely defined. Moreover, for all $y\in E^{\prime}$, $\delta^{*}(y|\int_{A} hM)=\int_{A} h\delta^{*}(y|M(\cdot))$. The map: $h\mapsto\int_{A} hM$ from $\mathcal{S}_{+}(T,\mathbb{R})$ to $\operatorname {cfb}(E)$ is uniformly continuous. Indeed, for all $f,g \in S_{+}(T;\mathbb{R})$, one has

$$\begin{aligned} \delta \biggl( \int_{A} fM, \int_{A} gM \biggr) = &\sup_{ \Vert y \Vert \leq1} \biggl\vert \int_{A}(f-g)\delta^{*}\bigl(y|M(\cdot)\bigr) \biggr\vert \\ \leq& \sup_{ \Vert y \Vert \leq1} \Vert f-g \Vert \bigl\vert \delta^{*} \bigl(y|M(A)\bigr) \bigr\vert \leq \Vert f-g \Vert \Vert M \Vert (T)< +\infty. \end{aligned}$$

Since $\mathcal{S}_{+}(T,\mathbb{R})$ is dense on $\mathcal {B}_{+}(T,\mathbb{R})$ and $\operatorname {cfb}(E)$ is a complete metric space, it has a unique extension to $\mathcal{B}_{+}(T,\mathbb{R})$: let $f\in\mathcal {B}_{+}(T,\mathbb{R})$ and let $(h_{n})$ be a sequence in $\mathcal {S}_{+}(T,\mathbb{R})$ converging uniformly to *f* on *T*; Therefore the integral $\int_{A} fM$ of *f* is uniquely defined by

$$\int_{A} fM=\lim_{n\rightarrow+\infty} \int_{A}h_{n}M. $$

Moreover,

$$\delta^{*}\biggl(y\Big| \int_{A} fM\biggr)= \int_{A} f\delta^{*}\bigl(y|M(\cdot)\bigr) $$

for all $y\in E^{\prime}$, $A \in\mathfrak{A}$ and for all $f\in \mathcal{B}_{+}(T,\mathbb{R})$. The map

$$\mathcal{B}_{+}(T,\mathbb{R})\rightarrow \operatorname {cfb}(E) \biggl(f\mapsto \int fM\biggr) $$

is additive, positively homogeneous, and uniformly continuous. If *m* is a vector measure defined on $\mathfrak{A}$, then the integral will be defined in the same manner. Denote $\mathcal{L}_{0}(\mathcal{B}(T,\mathbb{R}))$, $C^{h}(E^{\prime})$ the subspace of $\mathcal{L}(\mathcal{B}(T,\mathbb{R}), C^{h}(E^{\prime}))$ consisting of functions that verify the condition $l(f)\in C_{0}$ for all $f\in\mathcal{B}_{+}(T,\mathbb{R})$. □

### Lemma 3.3

*Let* $\mathcal{M}(\mathfrak{A},\operatorname {cfb}(E))$ *be the space of all bounded additive set*-*valued from* $\mathfrak{A}$ *to* $\operatorname {cfb}(E)$. *Let* $l\in\mathcal{L}_{0}(\mathcal{B}(T,\mathbb{R}), C^{h}(E^{\prime}))$. *Then there exists a unique set*-*valued measure* $M \in\mathcal {M}(\mathfrak{A}, \operatorname {cfb}(E))$ *such that* $l(f) = \delta^{*}(\cdot |\int fM)$ *for all* $f\in\mathcal{B}_{+}(T,\mathbb{R})$. *Conversely for all* $M \in\mathcal{M}(\mathfrak{A}, \operatorname {cfb}(E))$, *the mapping*: $f\mapsto\delta^{*}(\cdot |\int f^{+}M)-\delta^{*}(\cdot |\int f^{-}M)$ *from* $\mathcal{B}(T,\mathbb{R})$ *to* $C^{h}(E^{\prime})$ *is an element of* $\mathcal{L}_{0}(\mathcal{B}(T,\mathbb{R}), C^{h}(E^{\prime}))$. *Moreover*, $\Vert l \Vert = \Vert M \Vert (M)$.

### Proof

Let $l\in\mathcal{L}_{0}(\mathcal{B}(T,\mathbb{R}), C^{h}(E^{\prime}))$. Let us prove the uniqueness of the set-valued measure *M*. Assume that there exist two set-valued measures *M*, $M^{\prime}\in \mathcal{M}(\mathfrak{A}, \operatorname {cfb}(E))$ such that

$$\delta^{*}\biggl(\cdot\Big| \int fM\biggr)=l(f)=\delta^{*}\biggl(\cdot\Big| \int fM^{\prime}\biggr) $$

for all $f\in\mathcal{B}_{+}(T,\mathbb{R})$. Then, for all $A\in \mathfrak{A}$, $\delta^{*}(\cdot |\int1_{A}M)=l(1_{A})=\delta^{*}(\cdot |\int1_{A}M^{\prime}) (ie \delta ^{*}(\cdot |M(A))=\delta^{*}(\cdot |M^{\prime}(A)))$. Hence $M(A) = M^{\prime}(A)$ for all $A \in\mathfrak{A}$. Since $l\in \mathcal{L}_{0}(\mathcal{B}(T,\mathbb{R}), C^{h}(E^{\prime}))$ then *l* is associated with an additive, positively homogeneous and continuous set-valued map *L* from $\mathcal{B}_{+}(T,\mathbb{R})$ to $\operatorname {cfb}(E)$. Let $M : \mathfrak{A} \mapsto \operatorname {cfb}(E)$ be the set-valued map defined by $M(A) = L(1_{A})$ for all $A \in\mathfrak{ A}$. Then *M* is additive. It follows from the continuity of *L* that *M* is bounded. Moreover,

$$\int hM = L(h) $$

for all $h\in\mathcal{S}_{+}(T,\mathbb{R})$. Let $f \in\mathcal{B}_{+}(T,\mathbb{R})$ and let $(hn)$ be a sequence in $\mathcal{S}_{+}(T,\mathbb{R})$ converging uniformly to *f* on *T*. It follows from the definition of the integral $\int fM$ of *f* associated with *M* and the continuity of *L* that

$$L(f)=\lim_{n\rightarrow+\infty} L(h_{n})=\lim_{n\rightarrow+\infty} \in h_{n}(M)= \int fM. $$

Hence we have (Pan [23])

$$l(f) = \delta^{*}\biggl(\cdot\Big| \int fM\biggr) $$

for all $f\in\mathcal{B}_{+}(T,\mathbb{R})$. Conversely let $M\in\mathcal{M}(\mathfrak{A}, \operatorname {cfb}(E))$. Then the map $\theta: \mathcal{B}_{+}(T,\mathbb{R})\rightarrow C^{h}(E^{\prime})$ defined by

$$\theta(f)=\delta^{*}\biggl(\cdot\Big| \int f^{+}M\biggr)-\delta^{*}\biggl(\cdot\Big| \int f^{-}M\biggr) $$

verifies the condition $\theta(f)\in C_{0} $ for all $f\in\mathcal {B}_{+}(T,\mathbb{R})$. Let *j* be the isomorphism from $\operatorname {cfb}(E)$ to $C_{0}$ defined by $j(B) = \delta^{*}(\cdot B)$ and let *L* be the set-valued map from $\mathcal{B}_{+}(T,\mathbb{R})$ to $\operatorname {cfb}(E)$ defined by $L(f) =\int fM $ for all $f \in\mathcal{B}_{+}(T,\mathbb{R})$. Then *j* and *L* are continuous; therefore $\theta= j\circ L$ is continuous on $\mathcal {B}_{+}(T,\mathbb{R})$ and then on $\mathcal{B}(T,\mathbb{R})$. Let us prove now that $\Vert l \Vert = \Vert M \Vert (T)$. On the one hand, for all $y \in E^{\prime}$

$$\begin{aligned} \Vert l \Vert =&\sup_{ \Vert f \Vert \leq1} \bigl\Vert l(f) \bigr\Vert \\ \leq& \sup_{ \vert y \vert \leq1}\sup_{ \Vert f \Vert \leq1} \biggl\vert \delta^{*}\biggl(y\Big| \int f^{+}M\biggr)-\delta^{*}\biggl(y\Big| \int f^{-}M\biggr) \biggr\vert \\ \leq& \sup_{ \vert y \vert \leq1}\sup_{ \Vert f \Vert \leq1} \biggl\vert \int f^{+}\delta^{*}\bigl(y|M(\cdot)\bigr)- \int f^{-}\delta ^{*}\bigl(y|M(\cdot)\bigr) \biggr\vert \\ \leq& \sup_{ \vert y \vert \leq1}\sup_{ \Vert f \Vert \leq1} \biggl\vert \int f\delta^{*}\bigl(y|M(\cdot)\bigr) \biggr\vert . \end{aligned}$$

On the other hand we have

$$\Vert M \Vert (T)=\sup_{ \vert y \vert \leq1} \bigl\vert \delta^{*}\bigl(y |M(\cdot)\bigr) \bigr\vert (T). $$

Then it suffices to prove the equality $\sup_{ \Vert f \Vert \leq1} \vert \int f\delta ^{*}(y|M(\cdot)) \vert = \vert \delta^{*}(y|M(\cdot)) \vert (T)$, which is a classic result. □

## Main results and their proofs

### Theorem 4.1

*Let* *L* *be an additive*, *positively homogeneous and continuous set*-*valued map from* $\mathcal{B}_{+}(T,\mathbb{R})$ *to* $\operatorname {cfb}(E)$. *Then there is a unique bounded additive set*-*valued measure* *M* *from* $\mathfrak{A}$ *to* $\operatorname {cfb}(E)$ *such that*

$$L(f) = \int fM $$

*for all* $f\in\mathcal{B}_{+}(T,\mathbb{R})$. *Conversely for all bounded additive set*-*valued measure* $M : \mathfrak {A} \rightarrow \operatorname {cfb}(E)$, *the map*: $f\mapsto\int fM$ *from* $\mathcal{B}_{+}(T,\mathbb{R})$ *to* $\operatorname {cfb}(E)$ *is an additive*, *positively homogeneous and continuous set*-*valued map*.

### Proof

The second part follows from the definition of the integral with respect to *M*. Let $L : \mathcal{B}_{+}(T,\mathbb{R})\rightarrow \operatorname {cfb}(E)$ be an additive, positively homogeneous and continuous set-valued map and let

$$j : \operatorname {cfb}(E) \rightarrow C_{0}\bigl(B \mapsto j(B) = \delta^{*}(\cdot |B) \bigr). $$

We denote by *l* the unique extension of $j\circ L $ to $\mathcal{B}(T,\mathbb{R})$ for all $f\in\mathcal{B}(T,\mathbb {R})$, where

$$l(f) = j \circ L\bigl(f^{+}\bigr) - j \circ L\bigl(f^{-}\bigr) = \delta^{*}\bigl(\cdot |L\bigl(f^{+}\bigr)\bigr) - \delta ^{*}\bigl(\cdot |L\bigl(f^{-}\bigr)\bigr). $$

We have $l(f) = \delta^{*}(\cdot |L(f))\in C_{0}$ for all $f\in\mathcal {B}_{+}(T,\mathbb{R})$; then there exists a unique bounded additive set-valued *M* from $\mathfrak{A}$ to $\operatorname {cfb}(E)$ such that $l(f) = \delta^{*}(\cdot |\int fM)$ for all $f\in\mathcal{B}(T,\mathbb{R})$. Hence $L(f) =\int fM $ for all $f \in\mathcal{B_{+}}(T,\mathbb{R})$. □

The following corollary is partly known (see [16], Theorem 13, p.6).

### Theorem 4.2

*Let* $\mathcal{L}(\mathcal{B}(T,\mathbb{R}),E)$ *be the space of all continuous linear maps from* $\mathcal{B}(T,\mathbb{R})$ *to* *E* *and let* $\mathcal{M}(\mathfrak{A},E)$ *be the space of all bounded additive vector measures from* $\mathfrak{A}$ *to* *E*. *Let* $l \in\mathcal{L}(\mathcal{B}(T,\mathbb{R}),E)$. *Then there exists a unique vector measure* $m \in\mathcal{M}(\mathfrak{A},E)$ *such that* $l(f) =\int fm$ *for all* $f \in\mathcal{B}(T,\mathbb{R})$. *Conversely*, *given a vector measure* $m \in\mathcal{M}(\mathfrak {A},E)$, *the mapping* $f \mapsto\int fm $ *from* $\mathcal{B}(T,\mathbb {R})$ *to* *E* *is an element of* $\mathcal{L}(\mathcal{B}(T,\mathbb{R}),E)$. *Moreover*, $\Vert l \Vert = \Vert m \Vert (T)$.

### Proof

Put $\widetilde{E_{0}} = \{ \{x \}; x \in E \}$. Then $\widetilde{E_{0}}$ is a closed subspace of $\operatorname {cfb}(E)$. Let $j_{1}$ be the map from *E* to $\widetilde{E_{0}}$ defined by $j_{1}(x) = \{x \} $. Then $j_{1}$ is an isomorphism more a homeomorphism. Let $l^{\prime}$ be the restriction of $j_{1}\circ l$ to $\mathcal{B_{+}}(T,\mathbb{R})$. Then $l^{\prime}$ is additive, positively homogeneous and continuous. Therefore by Lemma 3.3 there exists a unique set-valued measure $m^{\prime}\in\mathcal{M}(\mathfrak{A}, \operatorname {cfb}(E))$ such that $l^{\prime}(f) =\int fm^{\prime}$ for all $f \in\mathcal {B_{+}}(T,\mathbb{R})$. It follows from this equality that $m^{\prime}(A)\in\widetilde{E_{0}}$ for all $A\in\mathfrak{A}$. Put $m = j_{1}^{-1}\circ m^{\prime}$. Then $m \in\mathcal{M}(\mathfrak {A};E)$ and verifies $m^{\prime}(A) = j_{1}(m(A))$ for all $A\in\mathfrak {A}$. We deduce that $\int fm^{\prime}= j_{1}(\int fm)$ for all $f \in\mathcal{B_{+}}(T,\mathbb {R})$; then $\int fm = j_{1}^{-1}\circ l^{\prime}(f) = l(f)$ for all $f \in\mathcal {B_{+}}(T,\mathbb{R})$ and consequently $l(f) =\int fm $ for all $f \in\mathcal{B}(T,\mathbb{R})$. The second part of corollary is proved as in Lemma 3.3. The equality $\Vert l \Vert = \Vert m \Vert (T)$ is a particular case of Theorem 4.1. □

By putting $E = \mathbb{R}$, we have the following result.

### Theorem 4.3

[23], Theorem 1, p.68

*Let* $\mathcal{M}(\mathfrak{A},\mathbb{R})$ *be the space of all bounded additive real*-*valued measures defined on* $\mathfrak{A}$. *Let* *l* *be a continuous linear functional defined on* $\mathcal{B}(T,\mathbb{R})$. *Then there exists a unique measure* $\mu\in\mathcal{M}(\mathfrak {A},\mathbb{R})$ *such that* $l(f) =\int f\,d\mu$ *for all* $f\in\mathcal{B}(T,\mathbb{R})$. *Conversely*, *for all measure* $\mu\in\mathcal{M}(\mathfrak{A},\mathbb {R})$, *the mapping*: $f \mapsto\int f\,d\mu$ *is a continuous linear functional defined on* $\mathcal{B}(T,\mathbb{R})$. *Moreover*, $\Vert l \Vert = \vert \mu \vert (T)$.

## Conclusions

In this paper, we discussed the Riesz integral representation for continuous linear maps associated with additive set-valued maps only using the existence of solutions for equilibrium equations with a Neumann type boundary condition. They inherited the advantages of the Shi-Liao type conjugate gradient methods for solving solutions for equilibrium equations with values in the set of all closed bounded convex non-empty subsets of any Banach space, but they had a broader application scope. Moreover, we also deduced the Riesz integral representation for set-valued maps, for the vector-valued maps of Diestel-Uhl and for the scalar-valued maps of Dunford-Schwartz (see [28]).
